# Automatic detection of foreign object intrusion along railway tracks based on MACENet

**DOI:** 10.1371/journal.pone.0329303

**Published:** 2025-08-06

**Authors:** Xichun Chen, Yu Tian, Ming Li, Bin Lv, Shuo Zhang, Zixian Qu, Jianqing Wu, Shiya Cheng

**Affiliations:** 1 School of Traffic and Transportation, Lanzhou Jiaotong University, Lanzhou, Gansu, China; 2 Department of Freight Transportation, Jinan Bureau Group Corporation, Jinan, Shandong, China; 3 School of Qilu Transportation, Shandong University, Jinan, Shandong, China; 4 Department of Science and Information, Lanzhou Bureau Group Corporation, Lanzhou, Gansu, China; Instituto Politecnico Nacional, MEXICO

## Abstract

Ensuring high accuracy and efficiency in foreign object intrusion detection along railway lines is critical for guaranteeing railway operational safety under limited resource conditions. However, current visual detection methods generally exhibit limitations in effectively handling diverse object shapes, scales, and varying environmental conditions, while typically incurring substantial computational overhead. To overcome these limitations, this study proposes a multi-level feature aggregation and context enhancement network (MACE-Net). The network architecture integrates the GOLD-YOLO module, an advanced object detection approach, alongside the updated deformable convolutional networks (DCNv3). The incorporation of DCNv3 allows the model to dynamically adapt its sampling positions according to actual object shapes, significantly enhancing feature extraction accuracy, especially for irregularly shaped intrusions. Additionally, the convolutional block attention module (CBAM) is employed to refine spatial and channel-wise feature representation, enabling the model to emphasize crucial object characteristics without substantially increasing computational complexity. Meanwhile, to improve localization robustness, the generalized intersection over union (GIoU) loss function is implemented, offering more reliable detection across various object sizes and shapes. Furthermore, to address the shortage of domain-specific datasets, we created a railway intrusion dataset comprising 7,200 images. Experimental results demonstrate that MACE-Net achieves superior detection performance, improving mAP@0.5 from 78.9% (baseline YOLOv8) to 83.8%—a notable increase of 4.9%. Meanwhile, the F1-score also rises by 5.2%. Importantly, despite significant accuracy gains, MACE-Net maintains computational efficiency similar to that of the baseline, affirming its suitability for real-time railway foreign object detection tasks under constrained energy and computational environments.

## 1 Introduction

Railway transportation—an essential pillar of a nation’s economic lifeline—directly affects public safety, property security, and overall economic stability. In recent years, as rail networks expand and train speeds increase, foreign-object intrusions along the tracks have emerged as a critical safety hazard [1]. Common examples include pedestrians trespassing, animals straying onto the rails, and rocks falling from adjacent slopes. Developing efficient and robust intelligent-monitoring systems to detect such intrusions is therefore of pressing practical importance. Traditional safeguards—manual patrols and physical barriers such as fences and warning signage—are hampered by delayed response times, limited coverage, and high operational costs. With rapid advances in artificial intelligence and computer vision, video-based intelligent-monitoring systems have become a prominent research focus. These systems can identify dynamic objects in complex environments in real time and issue precise early warnings, thereby markedly enhancing the safety and intelligence of railway operations [2].

Current technologies for detecting foreign objects along railways fall into three broad categories, each with its own strengths and shortcomings [3]. First, physical-sensor solutions detect intrusions by monitoring changes in physical signals and are largely insensitive to lighting conditions. Representative devices include infrared beams, lidar, and distributed vibration fiber-optic sensors. Nevertheless, adverse weather—such as rain or snow—can degrade their performance, leading to false alarms or omissions, and their deployment and maintenance costs remain high. Second, Radio Frequency Identification (RFID) pinpoints targets through tag–reader communication. Although effective for managing personnel and locating tagged assets in confined areas, RFID is ill-suited to the dynamic and untagged nature of most railway intrusions. Third, computer-vision approaches employ cameras to capture image or video streams and deep-learning algorithms for object detection and tracking. Vision-based methods provide non-contact monitoring, extensive spatial coverage, and continual performance gains through iterative training [4]. However, the heterogeneous conditions along railways—variable illumination, inclement weather, and vegetation movement—pose formidable challenges. Wind-driven foliage may be misclassified as moving objects, while rain or fog can obscure critical details, increasing the risk of missed detections. Furthermore, to satisfy real-time constraints on embedded edge devices, vision models must remain both accurate and lightweight—a demanding trade-off. Consequently, future research must reconcile detection performance, environmental robustness, and hardware efficiency [5].

In the field of railway foreign object detection, visual detection algorithms have undergone a technological evolution from traditional hand-crafted feature methods to deep learning approaches [6]. Traditional methods mainly rely on manually designed features and classifiers, with core processes including target detection, feature extraction, and classification. In target detection, commonly used techniques such as background modeling and optical flow methods are employed to effectively separate the foreground from the background. Feature extraction depends on hand-crafted features such as histogram of oriented gradients (HOG) for texture representation, local binary patterns (LBP) for local structure characterization, and Haar-like features, among others. Classification is typically performed using classifiers like support vector machines (SVM) and Random Forests to differentiate between foreign objects and interference. However, despite their excellent performance on small-scale datasets, traditional methods show limited robustness and adaptability when dealing with multi-scale targets, complex lighting, and dynamic backgrounds in railway scenarios [7].

With the advancement of deep learning technology, end-to-end detection frameworks based on convolutional neural networks (CNN) have gradually replaced traditional methods, offering the ability to automatically learn multi-level feature representations and perform end-to-end optimization. The mainstream deep learning algorithms include two-stage detection models such as Faster R-CNN [8], single-stage detection models represented by YOLO series and SSD [9], and lightweight network designs exemplified by MobileNet and ShuffleNet. These methods achieve a good balance between accuracy and speed, making them particularly suitable for real-time monitoring scenarios. In railway scenarios, significant progress has been made in the application of deep learning technologies, such as enhancing detection capabilities for small targets like fallen rocks through multi-scale feature fusion, reducing instantaneous interference false alarms by combining spatiotemporal context modeling, and improving algorithm generalization performance through data augmentation and synthesis. However, existing deep learning methods still face several challenges. On one hand, complex models are difficult to deploy on edge devices with limited computing power; on the other hand, false alarm rates are high in dynamic environments, especially in scenarios involving sudden changes in lighting and weather interference. Furthermore, due to the low frequency of railway foreign object intrusion events and the scarcity of annotated data, small sample learning capabilities are weak, which limits further development of the visual detection algorithms.

In summary, although current deep learning–based visual detection methods have achieved notable advances in accuracy, timeliness, and robustness, their core capability remains largely confined to answering whether a target has been detected. In railway safety management, however, the principal threat to train operation is not the mere presence of an object but whether it is located in, or about to enter, the track danger zone. Hence, the critical step for intelligent detection systems is to progress from simply “seeing an object” to “understanding the object’s semantic position.” Advancements in this direction not only improve the precision and efficiency of foreign-object detection but also strengthen the overall protective capacity of railway systems. Nevertheless, accurately determining whether a detected object lies within a hazardous railway area remains an urgent, unresolved challenge—one that is pivotal to the further enhancement of railway safety management.

In response to the aforementioned challenges, this paper presents an intelligent intrusion detection framework for complex railway environments, MACE-Net, with the following major contributions:

1. Integration of the GOLD-YOLO module, which utilizes a refined feature cascading and fusion strategy, enhances detection capabilities for various types of objects along the railway, particularly small and fast-moving objects, improving the model’s adaptability and response speed in complex scenarios.
2. A novel module, C2f_DCNv3, is introduced, incorporating the revised deformable convolution (DCNv3) with the C2f module. This integration optimizes the processing of image features via a CSP bottleneck structure enhanced with two convolution layers, significantly boosting the network’s capabilities in feature extraction and fusion within the YOLOv8n framework and enhancing detection of dynamic changes in foreign objects along railway environments.
3. The CBAM is integrated into the network’s backbone, enabling precise focus on key attributes of railway foreign objects, thereby elevating the precision of detection.
4. Development of a new dataset containing 7,200 images of railway line intrusions, covering a variety of real-world conditions across different weather and lighting situations, providing the model with extensive training and validation data.

The structure of this article is arranged as below. Section 2 briefly reviews related research in this field. Section 3 elaborates on the details of the proposed MACE-Net architecture. Section 4 introduces and describes the dataset employed in this study. Section 5 provides an in-depth analysis and discussion of experimental outcomes. Finally, Section 6 concludes the paper and summarizes key findings.

## 2 Related work

### 2.1 Object detection technology

Object detection technology has long been a cornerstone research topic in computer vision, continually garnering attention within the academic community [10]. Its core task is to recognize specific instances of certain categories (e.g., people, cars, bicycles, dogs, and cats) in images and determine their precise locations and extents [11,12]. Object detection is not only a pivotal stage in understanding image content but also underpins downstream vision tasks such as image segmentation, scene parsing, object tracking, and image captioning [13]. Broadly, object-detection techniques can be categorized into approaches based on manually engineered features and those driven by deep learning.

Traditional techniques primarily rely on carefully crafted features [13,14]. For example, the classic Viola–Jones (VJ) detector [15] applies a sliding-window strategy for object recognition, while the Histogram of Oriented Gradients (HOG) [15,16] strikes a balance between feature invariance and discriminability. The Deformable Part Model (DPM) further boosts detection precision through structural refinements [15]. Despite their practical merits, these approaches often exhibit limited accuracy and high computational cost.

The emergence of deep learning has radically altered the research landscape of object detection. AlexNet’s [17] triumph in the ImageNet competition was a landmark achievement for deep convolutional neural networks, heralding deep learning’s breakthrough in image recognition [18]. Thereafter, deep-learning-based detectors swiftly matured, stratifying into two primary paradigms: two-stage and one-stage. Two-stage methods first generate region proposals, which are then classified and accurately refined. Ren *et al*. [19,20] demonstrated that R-CNN and its derivatives markedly boosted performance through iterative refinements. One-stage methods, exemplified by the YOLO series, recast detection as a direct regression problem, markedly accelerating inference speed.

With ongoing technological progress, the YOLO algorithm has seen continual refinement, integrating state-of-the-art architectures and learning strategies, as in YOLOv5 and YOLOv8. These versions further improve speed and accuracy, particularly excelling at small-object detection [21]. Continuous innovation keeps the YOLO family at the forefront of real-time performance and accuracy, establishing it as a benchmark within the object-detection field [22].

### 2.2 Foreign object intrusion detection for railroads

In railway safety operations, accurately detecting foreign objects on the tracks is essential [23]. Current railway intrusion detection technologies can be broadly classified into contact and non-contact approaches. Contact-based detection typically employs sensors such as vibration fiber optics [7,24] and pulse electronic fences [25] installed on protective structures; these methods directly sense objects that touch the sensor. However, they are costly and cannot detect objects that do not physically contact the sensors—for example, main line sections extending more than 10 km often remain uncovered. By contrast, non-contact detection technologies, which rely on image recognition [26], radar, and similar means, provide more comprehensive monitoring of potential intrusions and can quickly alert drivers when anomalies are detected. Consequently, non-contact detection is gradually becoming the mainstream solution.

Among non-contact methods, deployment can be further divided into fixed detection devices installed beside the tracks and mobile detection devices mounted on onboard systems [27]. Although fixed installations can continuously monitor specific areas, they require extensive installation and maintenance efforts. By comparison, onboard camera systems can dynamically inspect conditions along the railway while the train is in motion, making them more economical and practical [28,29]. Nevertheless, both fixed and onboard solutions demand algorithms that remain robust under complex lighting as well as rain, snow, and fog conditions to meet real-time forward-looking requirements.

With the widespread application of deep learning technology in the field of computer vision, the detection of foreign object intrusions along railway lines has also seen significant advancements [29]. Specifically, the two-stage detection network based on Faster R-CNN offers high detection accuracy but lacks real-time performance [30]. For instance, Wang *et al*. [23] applied deep generative methods to the detection of foreign objects along railway lines, but the accuracy of detection still needs improvement. Wu *et al*. [31] proposed an intelligent detection method for defects in electric railway cotter pins based on an improved Faster R-CNN. However, the large scale of the model and poor real-time performance limit its deployment on mobile and embedded devices.

In contrast, single-stage detection networks, represented by YOLO, can achieve object detection with just one feature extraction, offering faster speeds compared to two-stage detection networks [32]. Wang *et al*. [33] addressed the issues of missed and false detections of small foreign objects against complex backgrounds along railway lines by proposing a high-accuracy recognition algorithm. This algorithm, by optimizing the backbone network, convolution kernels, and loss function of YOLOv5s and incorporating a weighted bidirectional feature pyramid network (BiFPN), significantly improves detection accuracy with minimal time cost. Dai *et al*. [34] introduced the YOLO-Former model based on improvements to YOLOv5, which incorporates visual transformers (ViT) and convolutional block attention modules (CBAM), designing a simple yet efficient method for detecting foreign objects along railway lines. These studies have achieved good recognition accuracy in foreign object detection. However, they mainly focus on detecting all objects within the scene, struggling to determine whether objects are in hazardous areas. Thus, while successful detection of foreign intrusions is crucial, it does not constitute the endpoint of railway safety.

## 3 The proposed network

### 3.1 Overall model of MACE-Net

**Fig 1:**
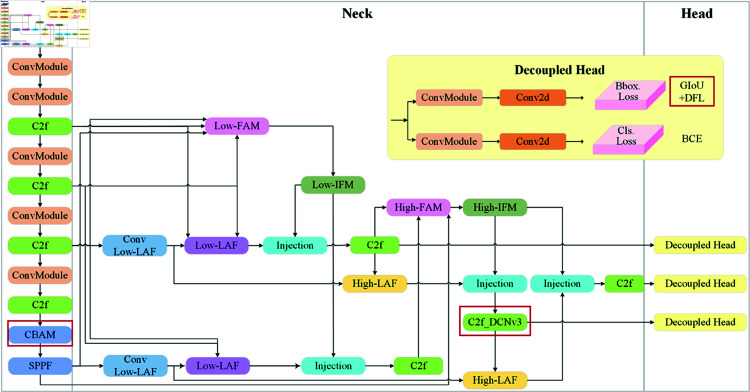
Multi-level feature aggregation and context enhancement network.

To address the frequent issues of missed detections and false alarms in foreign object intrusion detection tasks along railway lines, this paper proposes an improved deep learning model based on YOLOv8, termed multi-level feature aggregation and context enhancement network (MACENet). The structure of the proposed network is illustrated in Fig 1. By integrating multi-level feature aggregation and context enhancement strategies, MACENet significantly enhances the detection accuracy of small-sized and low-contrast foreign objects along railway tracks.

Specifically, the overall framework is divided into three stages: Backbone, Neck, and Head. At the end of the Backbone, a Convolutional Block Attention Module (CBAM) is inserted to perform channel and spatial attention, effectively suppressing redundant background information and highlighting potential intrusion features. This is followed by the use of a Spatial Pyramid Pooling Fast (SPPF) module to aggregate multi-scale receptive fields and provide unified global context priors for the entire network. In the Neck stage, the network constructs dual-path feature flows: shallow Low-LAF and deep High-LAF. These are selectively injected into the main pathway through multiple injection operations, achieving cross-scale complementation between fine-grained details and semantic information. To enhance the response to low-contrast small objects, a High-FAM module is added to deeper nodes. For improved geometric deformation modeling, the final C2f block in the Neck is upgraded to a deformable convolution version (C2f_DCNv3), enabling adaptive sampling of non-rigid intrusions such as gravel gaps and sleeper edges. Finally, at the Head stage, a fully decoupled detection head is adopted, separating the localization and classification branches. GIoU and DFL are jointly used for bounding box regression, while BCE is applied for object classification. This design ensures high localization accuracy while mitigating interference between tasks. The proposed model optimizes the processes of feature transmission and integration. It also effectively exploits contextual information through a spatial context-aware module, enabling more precise object detection within complex railway environments.

### 3.2 GOLD-YOLO module

In the past, the neck structure of the YOLO series utilized a traditional feature pyramid network (FPN) to achieve multi-scale feature fusion. This structure could only integrate features from adjacent layers, and other layers could only acquire features through recursive indirect access, resulting in significant loss of feature information during transmission. This limitation caused one layer’s information to only adequately assist adjacent layers, weakening its support for other global layers and limiting the overall effectiveness of information fusion. To avoid information loss during transmission in traditional FPN structures, this paper employs the GOLD-YOLO module to collect and fuse information at various levels, then distributes it across different levels. This approach avoids the inherent information loss associated with traditional FPN structures and enhances the partial information fusion capabilities of the neck without significantly increasing latency.

**Fig 2:**
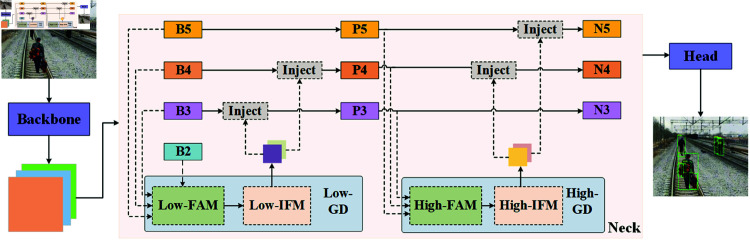
Architecture of GOLD-YOLO.

The GOLD-YOLO module, specifically designed for high-precision object detection in complex environments, is particularly suited for detecting foreign objects along railway lines. By integrating multi-level feature enhancement strategies, this module effectively handles various visual disturbances in railway environments, improving detection accuracy and efficiency. As shown in Fig 2, in the GOLD-YOLO module, the backbone network undertakes the core task of extracting features from the input image, generating multi-scale feature maps from B2 to B5. These feature maps, which contain information from basic visual details to high-level abstract features, lay the foundation for subsequent feature processing. The low-level feature augmentation module (Low-FAM) and the high-level feature augmentation module (High-FAM) then enhance the lower and higher-level feature maps, respectively. Low-FAM focuses on enhancing detail information to facilitate the detection of small or subtle foreign objects; High-FAM processes a broader range of contextual information, helping the system understand complex scenes and identify large foreign objects.

The feature injection mechanism, another core component of GOLD-YOLO, reintroduces the processed features from Low-FAM and High-FAM back into the main feature stream at multiple stages, including P3, P4, P5, N4, and N5. This approach allows the model to synthesize local details and global contextual information, significantly enhancing detection precision and robustness.

**Fig 3:**
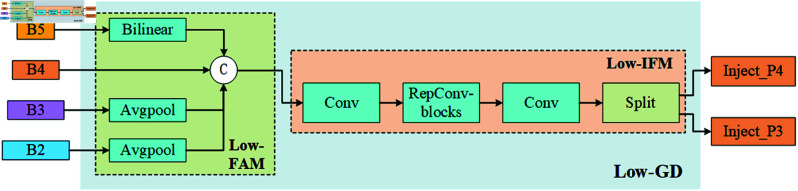
Low-stage gather-and-distribute branch.

**Fig 4:**
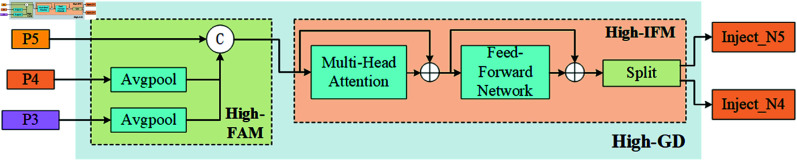
High-stage gather-and-distribute branch.

Additionally, GOLD-YOLO includes low-level and high-level gradient detectors (Low-GD and High-GD), as shown in Figs 3 and 4. These detectors are specially designed to capture minute changes and significant gradient information in the image, accurately locating and identifying potential foreign object intrusions along the railway lines. The neck module integrates all processed features to provide a comprehensive feature set for the final detection head, which utilizes these features for the ultimate object detection and localization.

Specifically, the Low-GD focuses on processing lower-level feature maps to capture rich texture information and edge features. This module is crucial for accurately delineating the contours of small or subtle foreign objects, especially on or near railway tracks. In contrast, the High-GD processes deeper feature maps, primarily identifying significant gradient changes and spatial transitions. This helps distinguish large objects and complex background structures in the scene. High-GD enhances the detection network’s ability to detect large foreign objects along railway lines, ensuring effective monitoring of critical areas in railway operation safety.

### 3.3 Deformable convolution DCNv3 module

In the task of detecting foreign objects along railway lines, traditional CNN, due to their fixed receptive fields and regular sampling patterns, struggle to effectively capture the diverse geometric variations of foreign object targets. Objects such as branches of trees or piles of stones along railway tracks often exhibit complex scale changes, shape deformations, and various postures due to different viewing angles. These feature variations pose significant challenges to detection tasks, reducing the accuracy of traditional convolutional methods. To more effectively capture the detailed features of foreign objects along railway tracks, this study introduces the deformable convolution module, which dynamically adjusts the sampling locations of the convolutional kernels, significantly enhancing the network’s adaptability to the morphological and positional changes of foreign objects.

**Fig 5:**
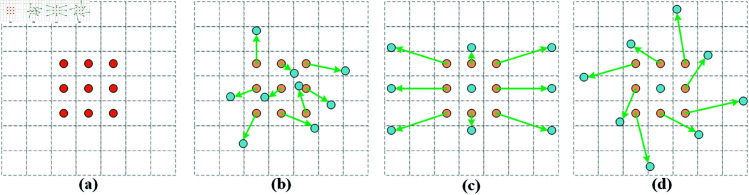
Standard convolution kernel and deformable convolution kernel.

Building upon DCNv1, Zhu *et al*. [35] proposed the more advanced DCNv2 algorithm. Unlike traditional convolution that uses fixed sampling points, deformable convolution introduces learnable offsets to the standard convolution, achieving a dynamic sampling mechanism that better captures geometric transformations of targets. Specifically, as shown in Fig 5, traditional convolution methods (Fig 5(a)) use a fixed regular grid for sampling operations. This grid is typically a pre-set rectangular area, and the convolutional kernel moves across the feature map with fixed strides, sampling features at fixed positions. This fixed pattern limits the receptive field of the convolution and its ability to capture irregular targets, making it inflexible to dynamic changes in object shapes. The computation of conventional convolution is expressed in Eq (1).

$$y\left(p_{0}\right)=\sum_{p_{i}\in C}\omega \left(p_{i}\right)\cdot x\left(p_{0}+p_{i}\right)$$ (1)

where *p*_0_ denotes the center of the input sample, while *p*_*i*_ specifies each sampling point within the sampling matrix *C*. The weight assigned to *p*_*i*_ is $\omega (p_{i})$. The input feature tensor at *p*_0_ is given by *x*(*p*_0_), and the convolution result at that location is denoted as *y*(*p*_0_).

Deformable convolution (Fig 5(b), 5(c), 5(d)), however, introduces an additional learnable offset that changes the positions of sampling points. This method allows each sampling point to adjust its position automatically based on the local information of the feature map during the convolution process, rather than being confined to a regular rectangular grid. In practice, the offset for each sampling position is dynamically learned and adapted by the neural network, making the sampling locations more aligned with the actual contours of the foreign objects. This sampling mechanism extends the effective receptive field of the convolutional kernel and flexibly captures local details of the target. Due to this dynamic and adaptive sampling method, deformable convolution can more effectively recognize objects with significant changes in scale, shape, and position, thereby significantly enhancing the model’s generalization ability and accuracy in detecting foreign objects in complex environments along railway lines. The computation of deformable convolution is expressed in Eq (2).

$$y\left(p_{0}\right)=\sum_{p_{i}\in C}\omega \left(p_{i}\right)\cdot x\left(p_{0}+p_{i}+△p_{i}\right)$$ (2)

where $△p_{i}$ denotes the offset positions.

**Fig 6:**
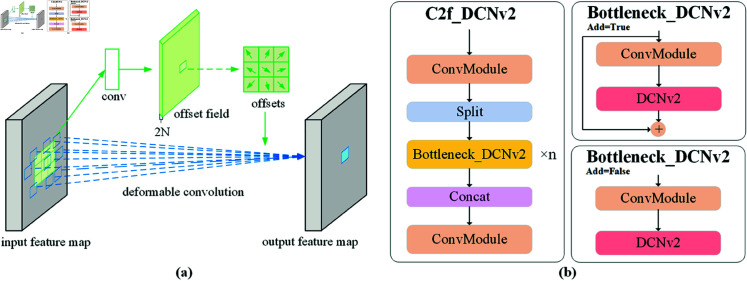
Schematic diagram of the structure perception module. (a) illustration of DCNv3; (b) illustration of C2f_DCNv3.

DCNv3 significantly enhances the model’s adaptability to complex image geometric transformations by introducing advanced spatial transformation capabilities and adaptive adjustment mechanisms. This version optimizes the computation of offsets and incorporates multi-scale and multi-angle offset controls. These enhancements allow the convolutional kernels to flexibly deform over a broader spatial range, capturing image features of varying scales and forms more accurately. As shown in Fig 6(a), DCNv3 first automatically adjusts the offset parameters through an enhanced learning module, which now responds to more variable image characteristics, such as edge curvature and object orientation. Subsequently, combined with a newly developed spatial attention module, the model weights the importance of each sampling point before executing the convolution, ensuring focus on the most informative areas of the image. This approach improves processing efficiency and significantly enhances the model’s robustness to environmental changes. It exhibits higher performance and adaptability across various detection and recognition tasks. The convolution process is given in Eq (3).

$$y\left(p_{0}\right)=\sum_{p_{i}\in C}\omega \left(p_{i}\right)\cdot x\left(p_{0}+p_{i}+△p_{i}\right)\cdot △m_{k}$$ (3)

where $△m_{k}$ represents the weight information.

However, merely utilizing the DCNv3 module is insufficient to significantly enhance the detection performance for foreign object intrusion along railway tracks. Therefore, this study proposes an improved module named C2f_DCNv3, based on the original YOLOv8 architecture, specifically targeting the C2f module within its neck structure, as shown in Fig 6(b). The proposed approach involves replacing the final C2f module (Level 21) in the YOLOv8 neck with the C2f_DCNv3 module. At this level, the output feature map P3 has a resolution of $\frac{1}{8}\times H\times W$, maintaining adequate spatial details for capturing the edges of small intruding objects such as cats, dogs, and gravel, while simultaneously integrating high-level semantic information from shallow and deep layers. Thus, it achieves an optimal balance between moderate spatial resolution and abundant semantic information.

DCNv3 performs deformation-aware sampling by learning pixel-level offsets. Its advantages can only be fully exploited when two conditions are simultaneously satisfied: (i) the feature maps have medium-to-high resolution capable of recovering object boundaries; and (ii) sufficient global semantics are available to guide offset prediction. Placing DCNv3 at Level 21 precisely fulfills these two prerequisites. In contrast, positioning DCNv3 earlier at the P2 layer ($\frac{1}{4}$ resolution) suffers from insufficient semantics, making it difficult to distinguish railway sleeper textures clearly. Conversely, deploying DCNv3 at deeper layers like P4 or P5 ($\frac{1}{16}$ or $\frac{1}{32}$ resolution) results in overly downsampled features, limiting the precision of offset estimation, and thus failing to achieve comparable performance. Consequently, introducing the C2f_DCNv3 module fully leverages DCNv3’s capability for modeling irregular object deformations while effectively controlling network complexity and computational overhead, ultimately realizing simultaneous improvements in both detection accuracy and real-time efficiency for foreign object intrusion along railway tracks.

### 3.4 Convolutional block attention module

To enhance the precision and reliability of foreign object detection along railway lines, we have decided to integrate an efficient attention mechanism module, convolutional block attention module (CBAM), into the YOLOv8 network. The CBAM module refines the channel and spatial features of images to effectively address the diversity in scale, shape, and orientation of foreign objects encountered in complex railway scenes. This module strengthens key foreign object features while maintaining computational efficiency, without requiring substantial additional computational resources. As a result, the detection system can precisely locate and identify potential hazards along the railway lines, significantly enhancing system response capability and recognition accuracy.

**Fig 7:**
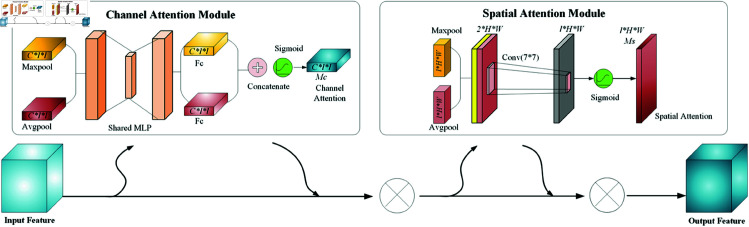
Architecture of CBAM.

CBAM is a modular and flexible attention mechanism that can be seamlessly integrated into any CNN architecture. By inserting CBAM blocks at any position within a CNN, the network gains the capability to adaptively focus on relevant features, both channel-wise and spatially. Fig 7 illustrates the architecture of the CBAM, a sophisticated attention mechanism designed to enhance the expressive power of convolutional neural networks. CBAM systematically refines feature maps by applying attention sequentially to the channel dimensions and then to the spatial dimensions, emphasizing important features and suppressing less relevant ones.

The channel attention module (CAM) of CBAM starts by processing the input feature maps through max pooling and average pooling operations to reduce the spatial dimensions, producing two separate channel descriptor vectors. These vectors compress the global spatial information of the feature maps and are then fed into a shared multi-layer perceptron (MLP) that contains two fully connected layers. The MLP processes each pooled feature vector separately to capture inter-channel dependencies. After processing by the MLP, the resulting feature vectors from both pooling methods are concatenated and passed through a sigmoid activation function. This produces a channel attention map *M*_*c*_, which provides a set of weights for each channel of the input feature map. By element-wise multiplying the channel attention map with the input feature map, the module selectively emphasizes informative features while suppressing less useful ones, thereby enhancing the network’s representational capability. The formulation of the channel attention module is shown in Eq (4):

$${𝐌}_{c}(𝐅)=\sigma (\mathrm{MLP}(\mathrm{AvgPool}(𝐅))+\mathrm{MLP}(\mathrm{MaxPool}(𝐅)))\;=\sigma \left({𝐖}_{1}\left({𝐖}_{0}\left({𝐅}_{\mathrm{Avg}}^{c}\right)\right)+{𝐖}_{1}\left({𝐖}_{0}\left({𝐅}_{\max}^{c}\right)\right)\right)$$ (4)

where *σ* denotes the Sigmoid activation function, ${𝐌}_{c}$ is the channel-attention feature map, and ${𝐖}_{0}$ and ${𝐖}_{1}$ are the MLP weights with ${𝐖}_{0}\in {\mathbb{R}}^{C/r\times C}$ and ${𝐖}_{1}\in {\mathbb{R}}^{C\times C/r}$.

After channel attention, the feature map is further refined through a spatial attention module (SAM), which focuses on the loci of attention within the feature map. SAM applies max pooling and average pooling along the channel dimension first, obtaining two two-dimensional maps that highlight significant features across spatial dimensions. These maps are concatenated and convolved with a 7×7 convolutional filter, effectively merging features to capture spatial dependencies. The convolution output is then processed through a sigmoid function, generating a spatial attention map *M*_*s*_. This map provides values between 0 and 1 for each spatial location, used to spatially scale the input feature map. Similar to the channel attention process, this spatial scaling allows the network to focus more on relevant spatial areas while diminishing others. The formulation of the spatial attention module is shown in Eq (5):

$${𝐌}_{s}(𝐅)=\sigma \left(f^{\;7\times 7}([\mathrm{AvgPool}(𝐅);\mathrm{MaxPool}(𝐅)])\right)\;=\sigma \left(f^{\;7\times 7}\left(\left[{𝐅}_{Avg}^{s};{𝐅}_{\max}^{s}\right]\right)\right)$$ (5)

where *σ* denotes the Sigmoid activation function, $f^{\;7\times 7}$ represents a 7 × 7 convolutional operation, ${𝐌}_{s}$ is the spatial-attention feature map, and ${𝐅}_{\text{Avg}}^{c}$ and ${𝐅}_{\text{max}}^{c}$ are the feature vectors generated by average-pooling and max-pooling operations, respectively.

### 3.5 Loss function

In applying the MACE-Net model for foreign object intrusion detection along railway tracks, we observed that the standard complete intersection over union (CIoU) loss exhibits certain limitations. Although the CIoU loss incorporates shape penalties, its intricate calculation increases training cost and processing time, making it less effective in scenarios involving offset or partially overlapping bounding boxes. These limitations are particularly noticeable in rapidly changing railway environments, negatively impacting the model’s responsiveness and accuracy.

**Fig 8:**
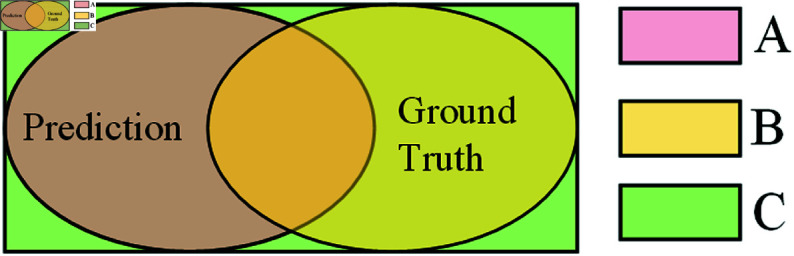
Diagram of IoU ratio.

To address these issues, we employed the generalized intersection over union (GIoU) loss as an alternative. GIoU loss simplifies the calculation by deducting the proportion of non-overlapping regions from traditional IoU metrics, thereby providing a more precise spatial relationship evaluation between predicted and ground-truth boxes. This computational simplicity enhances processing speed, reduces training complexity, and significantly improves detection accuracy. Furthermore, through its unique geometric adjustment method, GIoU adapts more effectively to complex railway scenarios involving objects of varying shapes and orientations. GIoU can be calculated as Eq (6):

$$GIoU=1-IoU+\frac{\left|C-(A\cup B)\right|}{\left|C\right|}$$ (6)

where IoU means the ratio of intersection to union. In the detection of foreign objects along railway tracks, it represents the overlap between the predicted and the actual bounding boxes, reflecting their relative accuracy. Formally, IoU can be computed as Eq (7):

$$IoU=\frac{\left|A\cap B\right|}{\left|A\cup B\right|}$$ (7)

where A represents the predicted area, B denotes the actual ground truth, and C indicates the smallest rectangle enclosing both A and B, as depicted in Fig 8.

In practical railway foreign object detection scenarios, models utilizing GIoU loss demonstrated superior performance and efficiency, confirming their effectiveness in real-world applications. This improvement notably enhances the model’s ability to accurately identify foreign objects within complicated railway environments, thus ensuring safer railway operations. Additionally, this modification substantially enhances both the reliability and operational efficiency of the detection system.

## 4 Dataset

Currently, there is no dedicated image dataset for foreign object intrusion detection in railway scenes. Existing public datasets, such as COCO, contain only a limited number of images focused on object detection in railway track or train-related scenes, and have yet to address foreign object detection in railway environments. As a result, models trained on these public datasets lack effectiveness in railway scene foreign object detection, struggling to learn track-specific features, leading to detection and missed detection issues.

To address this issue, this study collected data specifically for foreign object intrusion detection in railway scenes through multiple approaches and selected relevant samples from public datasets. Using data augmentation techniques, a foreign object intrusion dataset more suitable for railway scenarios was constructed. Firstly, regular surveillance videos were selected from the Lanzhou Railway Bureau’s video monitoring platform, covering various types of operational railway tracks, including ballast-based conventional passenger trains, freight railways, and ballastless high-speed railways. These videos included various scenes such as bridges, tunnel entrances, and tunnel interiors. Based on practical application requirements, diverse data under different meteorological conditions were collected from these surveillance videos. Secondly, we conducted field data collection along the railway, gathering video data from various time periods and weather conditions. After manual screening, a dataset consisting of 2,400 samples was constructed, with detailed annotations for each sample.

However, in actual railway lines, the data available for foreign object intrusion along the tracks is very limited, far from meeting the requirements for model training. To address this issue, we conducted a survey on common foreign object intrusion cases along railway tracks and communicated with railway inspection personnel and video surveillance staff. Based on these discussions, we identified several categories for training targets, including humans, obstacles (such as branches, falling rocks, etc.), various animals, cars, bicycles, and trains. Among these, pedestrians and various animals are the most common foreign objects along railway lines, particularly cows, sheep, cats, and dogs, which are more likely to invade the railway tracks. Due to their habits and activity ranges, these animals often appear near railways, especially in remote or open railway lines, where their presence poses a threat to the normal operation of the railway. Therefore, for these animals that are most prone to intruding onto the tracks, we obtained related data from public datasets. Then, using image processing techniques, we synthesized images of these animals with previously collected railway background samples.

Specifically, this study synthesizes animal foregrounds with railway backgrounds using a “Photoshop batch script + manual refinement” workflow. The procedure is as follows: animal images are first segmented in Photoshop 2024 using “Select Subject” and “Refine Edge,” with a 2-px feather and Content-Aware Fill applied to produce a smooth alpha mask. Next, a perspective grid is established in the railway scene image via “Vanishing Point,” and the animal is scaled and positioned according to the standard 1,435 mm track gauge to ensure consistent proportion and viewpoint. For lighting, the background’s primary light direction is estimated with a shadow vector; a soft shadow beneath the foreground is generated (Gaussian Blur σ = 8–12, opacity = 35–45%), and “Match Color” plus Curves adjustments in Lab space are used to harmonize brightness and color temperature. After synthesis, a random sample of 500 images underwent subjective evaluation; more than 90% were judged by three railway safety experts to be “virtually indistinguishable from real photographs.”

**Fig 9:**
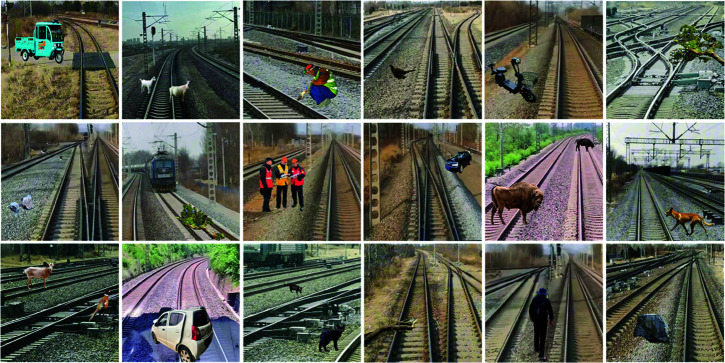
Illustration of the dataset.

As shown in Fig 9, through this approach, we generated a total of 7,200 railway scene samples with target categories, significantly expanding the diversity of the dataset. This method not only solved the issue of data collection but also provided a more comprehensive and enriched sample support for subsequent model training. Table 1 presents the statistical data for the six foreign object intrusion target categories. To ensure the rigor of the experiment and the reliability of the results, we made a reasonable division of the collected railway foreign object intrusion dataset. During the experimental process, the dataset was split into training, validation, and test sets in a 8:1:1 ratio. The three sets contain 5,760, 720, and 720 images of railway foreign object intrusion, respectively.

**Table 1:** Basic statistics of railway intrusion object categories.

Category	Person	Obsticle	Animal	Vehicle	Motor Bicycle
Number	1863	1032	3292	621	436

## 5 Experiments

### 5.1 Implementation details

All experiments are carried out under consistent conditions, and the specific experimental settings utilized are summarized in Table 2.

**Table 2:** Configuration of the experimental environment.

Experimental Environment	Detailed Information
CPU	Intel(R) Xeon(R) Silver 4316
GPU	NVIDIA GeForce RTX 3090
CUDA	11.8
System version	Rocky Linux 9.2 distribution
PyTorch	2.3.1
Python	3.8.12
Batch size	16
Epoch	300
Initial learning rate	$1\times 10^{-4}$
Min learning rate	$1\times 10^{-6}$
Optimizer	SGD

### 5.2 Evaluation metrics

In the task of railway foreign object intrusion detection, commonly used evaluation metrics include mean average precision (mAP), precision, recall, F1-score, and computational complexity metrics such as GFLOPs and Params. These evaluation metrics comprehensively assess the performance of models in different scenarios, especially in environments with complex lighting, dynamic backgrounds, and various interfering factors.

To elaborate further, mAP is a commonly used metric for evaluating object detection models, combining precision across different recall rates to provide an overall assessment. mAP considers the precision-recall balance at various thresholds and effectively evaluates the model’s overall performance across different classes. The calculation formula is Eq (8):

$$mAP=\frac{1}{N}\sum_{i=1}^{N}\int_{0}^{1}{\mathrm{Precision}}_{i}\left({\text{ Recall }}_{i}\right)d\left({\text{ Recall }}_{i}\right)$$ (8)

where N denotes the number of classes, and *Precision*_*i*_(*Recall*) represents the precision of the i-th class at a given recall. A higher mAP value indicates better overall performance across multiple classes and an ability to accurately identify and localize different intrusion objects.

Precision measures the proportion of true positive samples among all the samples predicted as positive by the model. High precision means that the majority of predicted positive samples are correct, helping reduce false positives. The formula for precision is Eq (9):

$$\text{ Precision }=\frac{TP}{TP+FP}$$ (9)

where TP is true positives, the number of correctly detected intrusion samples, and FP is false positives, the number of samples incorrectly detected as intrusions. Increasing precision helps minimize misdetection, enhancing the safety of railway operations.

Recall measures the proportion of true positive samples correctly identified out of all the actual positive samples. A high recall value indicates that the model can identify more of the actual intrusions, reducing missed detections. The formula for recall is Eq (10):

$$\text{ Recall }=\frac{TP}{TP+FN}$$ (10)

where FN is false negatives, representing missed intrusion samples. Improving recall is critical for ensuring railway safety, especially when dealing with dynamic backgrounds and complex lighting conditions.

The F1-score is the harmonic mean of precision and recall, providing a balanced evaluation that accounts for both precision and recall. The F1-score is especially useful for addressing class imbalance issues. A higher F1-score indicates that the model performs well in both precision and recall. The F1-score is calculated as Eq (11):

$$\text{ F1-score }=2\times \frac{\text{ Precision }\times \text{ Recall }}{\text{ Precision }+\text{ Recall }}$$ (11)

For railway foreign object intrusion detection, the F1-score helps assess the model’s overall performance, especially in handling imbalanced data. GFLOPs and Params are key metrics for evaluating the computational complexity and storage requirements of the model. GFLOPs represent the number of floating point operations required per inference, and Params denote the total number of trainable parameters in the model.

### 5.3 Results and discussion

**Fig 10:**
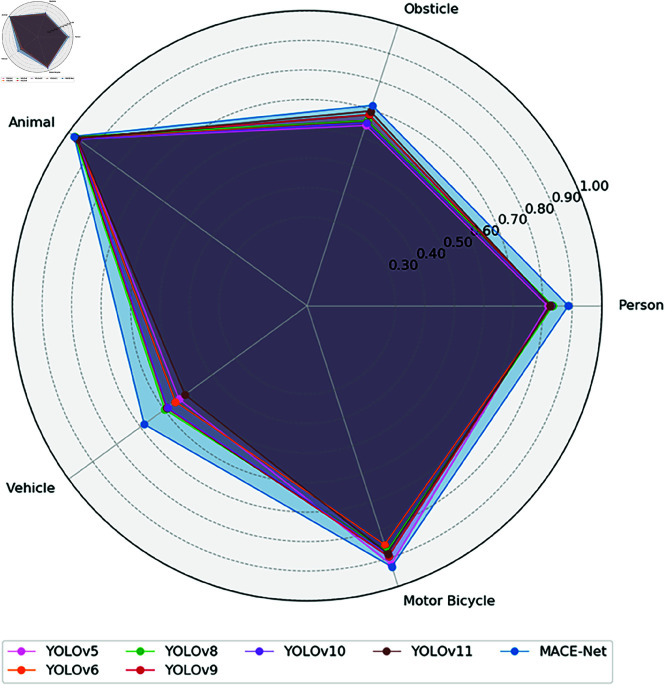
Performance comparison of mainstream detection models for railway intrusion detection.

To rigorously evaluate the enhanced performance of the proposed model, MACE-Net, for detecting foreign objects along railway lines, we conducted comprehensive comparative experiments with various versions of YOLO models, from YOLOv5 to YOLOv11. As indicated in Table 3 and Fig 10, the MACE-Net model significantly outperformed other methods in detecting various types of targets. The model achieved a mean average precision (mAP@0.5) of 83.9% across categories such as pedestrians, obstacles, animals, motor vehicles, and non-motor vehicles, demonstrating its superior generalization ability and effective target recognition performance in complex railway environments. Specifically, MACE-Net achieved substantial improvements in crucial detection categories for railway safety, with obstacle detection accuracy at 71.5% and pedestrian detection accuracy at 88.7%, highlighting its critical importance in practical applications. This is particularly important in practical application scenarios, further demonstrating MACE-Net’s outstanding generalization performance and detection capability across diverse categories of foreign object intrusion in railway environments.

**Table 3:** Comparison of intrusion detection accuracy across various models for railway scenarios.

Methods	AP@0.5					mAP@0.5
	Person	Obsticle	Animal	Vehicle	Motor Bicycle	
YOLOv5n	0.816	0.643	0.961	0.538	0.923	0.776
YOLOv6n	0.831	0.661	0.963	0.553	0.852	0.772
YOLOv8s	0.834	0.661	0.975	0.598	0.876	0.789
YOLOv9	0.825	0.681	0.968	0.589	0.894	0.791
YOLOv10	0.827	0.652	0.962	0.587	0.884	0.782
YOLOv11	0.826	0.695	0.964	0.513	0.884	0.776
**MACE-Net**	0.887	0.715	0.978	0.684	0.931	**0.839**

Additionally, the comprehensive performance comparison in Table 4 confirms that MACE-Net substantially exceeds other YOLO series models in detection performance along railway lines. This superiority is particularly evident in precision metrics (mAP@0.5 at 0.839) and overall performance indices such as the F1-score of 0.834, indicating clear advantages in both precision and recall. However, these performance gains come with an increase in model parameters (13.4M Params) and computational complexity (29.6 GFLOPs), which are significant compared to lighter YOLO models, such as YOLOv11 with only 2.6M parameters and 6.3 GFLOPs. Despite this, considering the high sensitivity to accuracy and reliability in railway foreign object detection, the benefits in detection stability and accuracy from MACE-Net far outweigh the costs of increased computational resources, proving its higher application value in actual railway safety monitoring scenarios. Meanwhile, in terms of inference speed, MACE-Net maintains near-real-time efficiency comparable to the baseline model, achieving 43 frames per second (fps) in actual tests, which is only 4 fps lower than YOLOv8s at 47 fps. This result clearly demonstrates that the proposed approach significantly improves detection accuracy while simultaneously meeting the stringent real-time requirements of railway field applications, thereby achieving breakthroughs in both precision and efficiency.

**Table 4:** Comparison of computational efficiency and robustness of different models.

Methods	mAP@0.5	Params/M	GFLOPs	F1-Score	FPS
YOLOv5n	0.776	2.5	7.1	0.785	143
YOLOv6n	0.772	4.2	11.8	0.787	86
YOLOv8s	0.789	11.1	28.4	0.782	47
YOLOv9	0.791	2.0	7.6	0.784	134
YOLOv10	0.782	2.3	6.5	0.780	156
YOLOv11	0.776	2.6	6.3	0.761	161
**MACE-Net**	0.839	13.4	29.6	0.834	43

**Fig 11:**
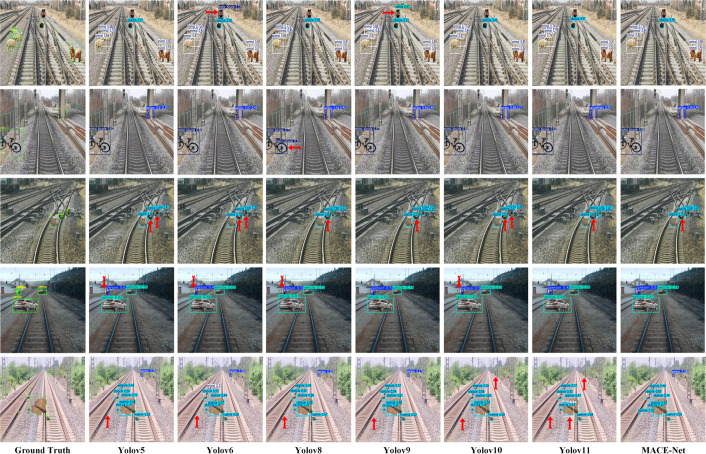
Visualized railway intrusion detection results of comparative models.

Moreover, as illustrated in Fig 11, visualizing the detection results of foreign objects along railway lines more directly showcases the performance differences between models. The visualization clearly demonstrates that MACE-Net, compared to other YOLO models, achieves significantly higher detection accuracy under complex background conditions and diverse target types typical of railway environments. MACE-Net excels particularly in identifying distant small-scale targets, such as pedestrians and animals far away, and various obstructions like bicycles on complex tracks, with fewer missed detections and false positives. In contrast, earlier and other versions of YOLO models often fail to detect these detailed targets adequately, as highlighted by red arrows in the visualization images, especially for distant, small-sized foreign objects.

**Fig 12:**
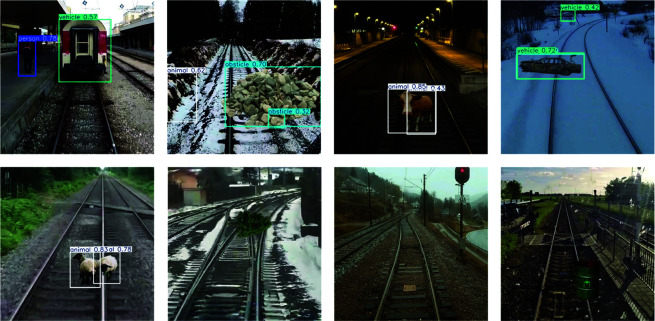
Examples of false-positive and false-negative detections.

Although MACE-Net achieves commendable overall detection performance, it inevitably encounters issues of false positives and false negatives in complex real-world scenarios, thereby affecting the system’s stability and reliability. As illustrated in Fig 12, examples of such false positives and false negatives are presented. Specifically, false positives primarily occur in scenes with significant background interference or when targets closely resemble the background. For instance, strong winds causing vegetation alongside tracks to sway substantially or shadows and bright spots generated under intense lighting conditions can be misinterpreted by the model as moving foreign objects. Additionally, highly reflective or structurally similar background elements may also trigger incorrect detections. Conversely, false negatives typically occur when the targets exhibit indistinct features, low color contrast, or partial occlusion. Examples include small animals near sleepers under low-light conditions at night, foreign objects with colors similar to rail surfaces, or partially obstructed intrusions behind rail structures. Such missed detections are particularly critical in safety monitoring as they often involve high-risk targets. These issues highlight areas for improvement in the current model’s environmental adaptability, identification of marginal targets, and contextual understanding.

In conclusion, although MACE-Net has slightly higher parameter and computational costs, it exhibits superior performance in the critical task of detecting foreign objects along railway lines. Its enhanced detection accuracy and robustness better meet the real-time safety monitoring requirements of railway environments, making MACE-Net a preferable choice over other versions of the YOLO series in applications prioritizing detection performance and reliability along railway lines.

### 5.4 Ablation study

Based on the ablation study results shown in Table 5, the effectiveness of key modules in the MACE-Net for railway intrusion detection tasks was rigorously validated and analyzed.

**Table 5:** Results of ablation studies on railway intrusion detection.

Models	Level 15	Level 18	Level 21	mAP@0.5	F1-Score	Params/M	GFLOPs
Baseline				0.789	0.782	11.1	28.4
+C2f_DCNv3	✓			0.789	0.782	11.1	28.1
		✓		0.794	0.787	11.0	28.1
			✓	0.813	0.804	10.7	28.1
	✓	✓		0.798	0.793	11.0	27.8
	✓		✓	0.795	0.790	10.7	27.8
		✓	✓	0.794	0.790	10.6	27.7
	✓	✓	✓	0.805	0.798	10.5	27.4
+CBAM				0.796	0.791	11.4	28.4
+Gold-YOLO				0.799	0.792	13.6	29.9
+GIoU_loss				0.791	0.784	11.1	28.4
+All			✓	0.838	0.834	13.4	29.6

* The symbol “” denotes the inclusion of a module in the baseline network.

Initially, YOLOv8 was used as the baseline model with an mAP@0.5 of 0.789 and an F1-score of 0.782, featuring parameters (Params) of 11.1M and a computational complexity (GFLOPs) of 28.4. Tests were conducted by replacing the C2f_DCNv3 module at different network levels (Level 15, Level 18, Level 21). Results indicated no change in mAP and F1-score at Level 15 (both remaining at 0.789 and 0.782 respectively), with a slight decrease in GFLOPs to 28.1. At Level 18, mAP increased to 0.794 (an improvement of 0.5%), and F1-score to 0.787 (an improvement of 0.5%), with a reduction in parameters to 11.0M and GFLOPs to 28.1. Significant performance enhancements were observed at Level 21, with mAP and F1-score reaching 0.813 and 0.804, respectively, up by 2.4% and 2.2% from the baseline, while parameters and computational complexity further reduced to 10.7M and 28.1 GFLOPs.

Moreover, testing combinations of different C2f_DCNv3 levels showed that combining replacements (Level 15+18, Level 15+21, Level 18+21, Level 15+18+21) all enhanced model performance. Particularly, replacing all three levels simultaneously (Level 15+18+21 combination) achieved an mAP of 0.805, up 1.6% from the baseline, and an F1-score of 0.798, with parameters reduced to 10.5M and GFLOPs to 27.4.

Further assessments were made on other modules’ contributions independently. Introducing the CBAM attention module alone on the baseline improved mAP to 0.796 and F1-score to 0.791, albeit with a slight increase in parameters and computational demand. Adding the Gold_YOLO module alone resulted in mAP reaching 0.799 and F1-score rising to 0.792, but significantly increasing parameters to 13.6M and computational complexity to 29.9 GFLOPs. Replacing the loss function with GIoU_loss alone, mAP and F1-score were 0.791 and 0.784 respectively, showing performance improvements with minimal change in parameters and computational demand.

Lastly, integrating all modules (C2f_DCNv3, CBAM, Gold_YOLO, and GIoU_loss) formed the MACE-Net model. This model achieved the best detection performance with an mAP of 0.838 (an increase of 4.9% over the baseline) and an F1-score of 0.834 (an increase of 5.2%), maintaining a total parameter count of 13.4M and a computational complexity of 29.6 GFLOPs. These results demonstrate a significant synergistic effect between the modules, markedly enhancing accuracy and robustness in railway intrusion detection tasks while balancing model complexity and computational efficiency.

## 6 Conclusion

Given the significant safety risks and operational impacts posed by foreign object intrusions along railway tracks, developing accurate and efficient intrusion detection systems is critical. However, balancing detection accuracy and real-time efficiency remains challenging in practical railway scenarios, particularly in mobile or resource-constrained monitoring devices. To address these issues, this paper proposes a multi-level feature aggregation and context enhancement network (MACE-Net). First, the proposed C2f_DCNv3 module utilizes advanced deformable convolutions, effectively capturing variable spatial features of foreign objects, substantially enhancing detection capabilities for irregularly shaped or distant intrusions. Next, an integrated convolutional block attention module (CBAM) adaptively highlights critical visual information about objects along railway lines, significantly improving feature representation. Additionally, integrating the GOLD-YOLO module enhances multi-scale information aggregation, enabling efficient identification of objects in complex environments. Finally, incorporating the generalized intersection-over-union (GIoU) loss accelerates model optimization and reduces localization errors, boosting detection robustness.

Rigorous evaluation using real railway datasets shows that MACE-Net markedly outperforms traditional YOLO-series models in railway intrusion detection tasks. Compared to the YOLOv8 baseline, MACE-Net achieved a 4.9% improvement in mAP@0.5 and a 5.2% increase in F1-score, with notable advantages in detecting pedestrians and small obstacles. Despite an increase in model complexity and parameters, the substantial gains in critical object detection accuracy validate the practical applicability and reliability of the proposed model in real railway environments.

Future research will explore lighter-weight network structures and multi-task learning strategies to reduce computational overhead. Moreover, integrating multimodal data fusion and anomaly behavior recognition techniques will also be studied to realize a more accurate and comprehensive railway safety protection framework, ultimately contributing robust technical support for advancing railway intelligent safety monitoring technologies.
